# Nonlinear localized modes in one-dimensional nanoscale dark-state optical lattices

**DOI:** 10.1515/nanoph-2022-0213

**Published:** 2022-06-22

**Authors:** Zhiming Chen, Jianhua Zeng

**Affiliations:** State Key Laboratory of Transient Optics and Photonics, Xi’an Institute of Optics and Precision Mechanics of Chinese Academy of Sciences, Xi’an 710119, China; School of Science, East China University of Technology, Nanchang 330013, China; Collaborative Innovation Center of Light Manipulations and Applications, Shandong Normal University, Jinan 250358, China; University of Chinese Academy of Sciences, Beijing 100049, China

**Keywords:** Bose–Einstein condensation, gap solitons, nanoscale dark-state optical lattices, three-level atomic system

## Abstract

Optical lattices (OLs) with conventional spatial periodic *λ*/2, formed by interfering the counterpropagating laser beams with wavelength *λ*, are versatile tools to study the dynamical and static properties of ultracold atoms. OLs with subwavelength spatial structure have been realized in recent quantum-gas experiment, offering new possibility for nonlinear and quantum control of ultracold atoms at the nano scale. Herein, we study theoretically and numerically the formation, property, and dynamics of matter-wave localized gap modes of Bose–Einstein condensates loaded in a one-dimensional nanoscale dark-state OL consisted of an array of optical subwavelength barriers. The nonlinear localized modes, in the forms of on- and off-site fundamental gap solitons, and dipole ones, are demonstrated; and we uncover that, counterintuitively, these modes exhibit always a cusplike (side peaks) mode even for a deeply subwavelength adiabatic lattice, contrary to the previously reported results in conventional deep OLs where the localized gap modes are highly confined in a single lattice cell. The (in)stability features of all the predicted localized modes are verified through the linear-stability analysis and direct perturbed simulations. Our predicted results are attainable in current ultracold atoms experiments with the cutting-edge technique, pushing the nonlinear control of ultracold atoms with short-period OLs as an enabling technology into subwavelength structures.

## 1 Introduction

Because of possessing unique features (e.g. size, strength and structure) that are tunable, controllable and manageable freely, optical lattices (OLs) become a chosen multifunctional tool for understanding and manipulating the dynamical and stationary properties of quantum gases [1–4] whose inter-particle interactions, per se, can be changed at will by means of Feshbach resonances [5–8]. Specific highlights include but not limit to the creation of Bose–Einstein condensation (BEC) by laser cooling [9], the realizations of quantum simulators with phase transition between superfluid and Mott-insulator [10–13], synthetic gauge fields and spin–orbit coupling [14, 15], new topological quantum materials [16, 17], as well as new-generation atomic clocks with unprecedented precision [18, 19]. OLs are optical periodic potentials fabricated by the interference of pairs of counterpropagating laser beams with wavelength *λ* and, therefore, featuring a half wavelength characteristics—the lattice period always equates *λ*/2, because of which, manipulating quantum matters using the regular OLs [1], [2], [3], [4, 20], [21], [22], [23], [24], [25], [26] (and other periodic structures in optics context [27–33]) has been exclusively confined to the wavelength scale (i.e., few hundred nanometers).

In past years, several techniques can contribute to subwavelength lattice structures, including adiabatic optical potentials with *λ*/4 periodicity induced by Raman coherences [34], sculpting a subwavelength lattice potential using multiphoton transitions [35–37], radio-frequency-dressed state-dependent subwavelength lattices [38, 39], dynamic spin-dependent lattices (of subwavelength spacing) with a time-periodic modulation [40], dark state optical potentials with a subwavelength structure [41–45]. In particular, recent experimental observation has confirmed the creation of dark state OLs with subwavelength optical barriers (resemble an optical “Kronig–Penney” potential) with width less than *λ*/50 = 10 nm [43]. Subwavelength lattice structures provide new opportunities for investigating both the weakly interacting single-particle (mean-field theory) property and strongly interacting quantum many-body physics of ultracold quantum gases (including ultracold polar molecules [46]) down to the scale of tens of nanometers, such as enhancing the whole energy scale; greatly facilitating the formation of low-temperature states; modulating the balanced (atom–atom) interactions between on-site and neighboring atoms [41–43]; engineering artificial gauge fields [40]; performing subwavelength quantum nondemolition measurements [47, 48]; imaging of atoms using nanoscale atomic density microscopy with unprecedented spatial resolution approximately (breaking the diffraction limit) [49]; etc.

By adopting the above-mentioned one-dimensional (1D) optical “Kronig–Penney” potential [42, 43] in which Bose–Einstein condensates (BECs) are loaded, we report on theoretical and numerical studies of the formation, property, and dynamics of nonlinear self-trapping of matter waves, representing as fundamental gap solitons of on-site and off-site types, as well as the dipole ones, found in the first, second, and third finite band gaps of the associated linear Bloch spectrum, in such 1D setting with both shallow and deep lattice depths. In particular, we uncover that these self-trapped modes exhibit always a cusplike (side peaks) characteristic even for a deeply nanoscale dark-state lattice, different from the scenario of conventional deep OLs where no any modulation exists for localized gap modes. The (in)stability features of all the self-trapped modes are verified by linear-stability analysis and direct perturbed simulations. Our findings provide in-depth insights into soliton physics in periodic systems, pushing nonlinear manipulation of gap solitons into periodic potentials on the subwavelength scale. Considering the fact that the 1D Bose–Einstein gap solitons and broad gap waves of ^87^Rb atoms with repulsive atom–atom interaction have, respectively, been observed in weak (shallow) [50] and superimposed deep [51] optical periodic potentials, there is no doubt that the localized gap solitons predicted here are observable in such experiments but with nanoscale dark-state optical “Kronig–Penney” potentials.

## 2 Theoretical model

### 2.1 Nonlinear Schrödinger equation

Dynamics of wave function Ψ(*x*, *t*) of the BECs in a 1D nanoscale dark-state OL consisted of an array of optical subwavelength barriers can be described by mean-field theory, Gross–Pitaevskii equation (nonlinear Schrödinger equation):

(1) $$i\hbar \frac{\partial \Psi }{\partial t}=-\frac{\hbar^{2}}{2m}\frac{\partial^{2}\Psi }{\partial x^{2}}+E_{R}V\left(x\right)\Psi +\frac{4\pi \hbar^{2}a_{s}}{m}{\left|\Psi \right|}^{2}\Psi .$$

**Figure 1:**
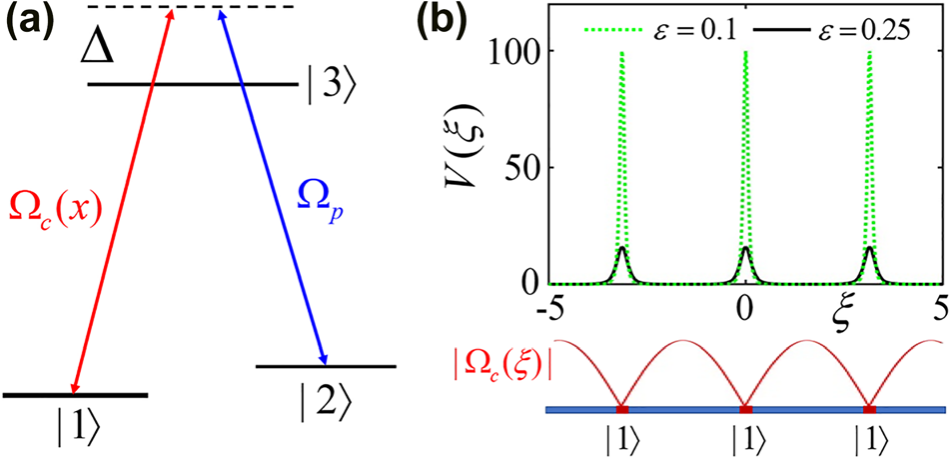
Physical scheme for creating a nanoscale dark state optical lattice. (a) The optical subwavelength barrier is created under a three-level (Λ-type) atomic system that exists a “dark state” as supposition of two lowest atomic (ground) states |1⟩ and |2⟩, which are connected by a resonant Raman coupling between a strong control field Ω_c_(*x*) and a weak probe field Ω_p_ (see text). (b) The spatial (geometry) structure of the generated subwavelength dark state optical lattice with different ratio of Rabi frequencies *ɛ* (=Ω_p_/Ω_c_).

Here *E*_R_ = *ℏ*^2^*k*^2^/*m*, with *m* the mass of atoms and *k* = 2*π*/*λ*, *λ* being the wavelength of light mentioned above. The last (defocusing nonlinear) term accounts for atoms with repulsive–repulsive interactions, with defocusing scattering length of atoms *a*_s_ > 0. We stress that our potential *V*(*x*) is the same as the nanoscale dark-state optical “Kronig–Penney” potential proposed in [42] and demonstrated experimentally in [43], more relevant details can be read from there. We briefly describe the formation of such novel optical potential: the optical subwavelength barrier of the dark-state lattice potential is prepared in a three-level (Λ-type) atomic system which owns a “dark state” as supposition of two lowest atomic (ground) states |1⟩ and |2⟩ whose connection, in reality, is bridged by a resonant Raman coupling between a strong control field Ω_c_(*x*) = Ω_c_sin(*kx*) and a weak probe constant field Ω_p_, see Figure 1(a). Thus, the nanoscale dark-state OL *V*(*x*), in essence, is emerged from the mechanism of nonadiabatic corrections to adiabatic Born–Oppenheimer potentials for atomic motion [41, 42]. Optical periodic potential with a subwavelength structure yields:

(2) $$V\left(x\right)=\frac{\cos^{2}\left(kx\right)}{\varepsilon^{2}{\left[1+\varepsilon^{-2}\sin^{2}\left(kx\right)\right]}^{2}}.$$

Here *ɛ* = Ω_p_/Ω_c_ denotes the ratio of Rabi frequencies. The nanoscale dark-state optical potential *V*(*x*) can be viewed as optical “Kronig–Penney” potential provided that *ɛ* ≪ 1 [52–54], according to Figure 1(b). It is also observed that a decrease of *ɛ* would render the dark-state lattice change from shallow depth to deep one (lattice strength). *V*(*x*) is a periodic arrangement of narrow potential barriers spaced by *λ*/2, with the barrier heights scaling as 1/*ɛ*^2^, where the energy levels |1⟩, |2⟩, and |3⟩ can be selected, respectively, as 5^2^*S*_1/2_(*F* = 1), 5^2^*S*_1/2_(*F* = 2), and 5^3^*P*_1/2_(*F* = 2) of ^87^Rb atoms [55]. Therefore, the wavelength of light is around 795 nm, and the full width at half maximum is about tens of nanometers or less.

To discuss conveniently, we convert Eq. (1) into the dimensionless form:

(3) $$i\frac{\partial \psi }{\partial \tau }=-\frac{1}{2}\frac{\partial^{2}\psi }{\partial \xi^{2}}+V\left(\xi \right)\psi +{\left|\psi \right|}^{2}\psi ,$$

where the scales of coefficients are *τ* = *tE*_R_/ℏ, *ξ* = *kx*, and $\psi =2k^{-1}\sqrt{\pi a_{R}}\Psi$ .

We search stationary wave function $\psi \left(\xi ,\tau \right)=\phi \left(\xi \right){\text{e}}^{-\text{i}\mu \tau }$ (chemical potential *μ*) for Eq. (3), the underlying stationary equation yields:

(4) $$\mu \phi =-\frac{1}{2}\frac{\partial^{2}\phi }{\partial \xi^{2}}+V\left(\xi \right)\phi +{\left|\phi \right|}^{2}\phi .$$

In the following, the soliton solutions *ϕ*(*ξ*) are constructed by choosing a properly selected initial Gaussian guess from Eq. (4) via Newton’s interaction, their linear stability is evaluated through linear stability analysis, and direct perturbed simulation of the dynamical Eq. (3) by means of the fourth-order Runge–Kutta method. The methods are expressed elaborately in Appendix A. To facilitate discussion, the number of ultracold atoms *N* (norm) is defined as *N* = ∫|*ϕ*|^2^d*ξ*.

### 2.2 Band-gap structures of the nanoscale dark-state lattice

**Figure 2:**
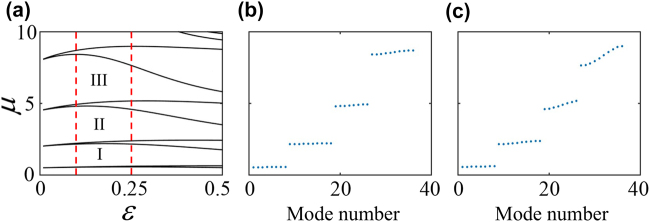
Band-gap structure of the 1D nanoscale dark state optical lattice. (a) Linear Bloch-wave spectrum with varying *ɛ*. (b, c) Eigenvalues of linear Bloch-wave modes forming a band-gap structure at *ɛ* = 0.1 and *ɛ* = 0.25. The first three (first, second, and third) finite band gaps are denoted by I, II, and III, respectively.

Before going deep insight into the gap solitons supported by the full model, one should know clearly about the band-gap structure of the underlying linear model. By discarding the last term of Eq. (4), and by solving the eigenvalue problem, we can get the associated linear-Bloch spectrum as a function of *ɛ* (the ratio of Rabi frequencies Ω_*p*_/Ω_*c*_) which is depicted in Figure 2(a). Recall that for increasing *ɛ* leads to decrease the lattice strength [c.f. Figure 1(b)], making the width of finite band gaps shrinks gradually [see Figure 2(a)]. For particular values of *ɛ* = 0.1 and *ɛ* = 0.25, representing the nanoscale dark-state lattice with deep strength and the shallow one, the corresponding eigenvalues of linear Bloch-wave modes forming a band-gap structure are displayed, respectively, in Figure 2(b) and (c). It is seen from both panels that there are first three finite band gaps (the first, second and third one), within where the matter-wave gap solitons may be resided. An unique feature of the band-gap structure of a deeply nanoscale dark-state lattice is the smoothness of the lowest Bloch bands, according to Figure 2(b) at *ɛ* = 0.1 which corresponds to a very deep lattice with strength 100 (c.f. Figure 1(b)), flat Bloch bands appear at around *ɛ* = 0.03 [see Figure 2(a)] while the lattice strength is 1000, amazingly deep.

An extraordinary property of the bang-gap structure of the nanoscale dark-state lattice is that the widths of higher gaps are wider than that of lower gaps [comparing I, II, III regions in Figure 2(a)–(c)], which challenges our knowledge of conventional periodic potentials (including the “Kronig–Penney” potentials [52–54]) where the widest width is always for the first band gap. Noticeably, the latter fact is followed up for dark-state lattice if the *ɛ* ≥ 0.4 [see Figure 2(a)], since under such a situation the dark-state lattice recovers to a conventional OL which, as pointed out elsewhere [1–4], can be more easily to be created without using the dark-state of the atoms.

## 3 Numerical results

### 3.1 Fundamental gap solitons of on- and off-site types in both shallow and deep lattices

**Figure 3:**
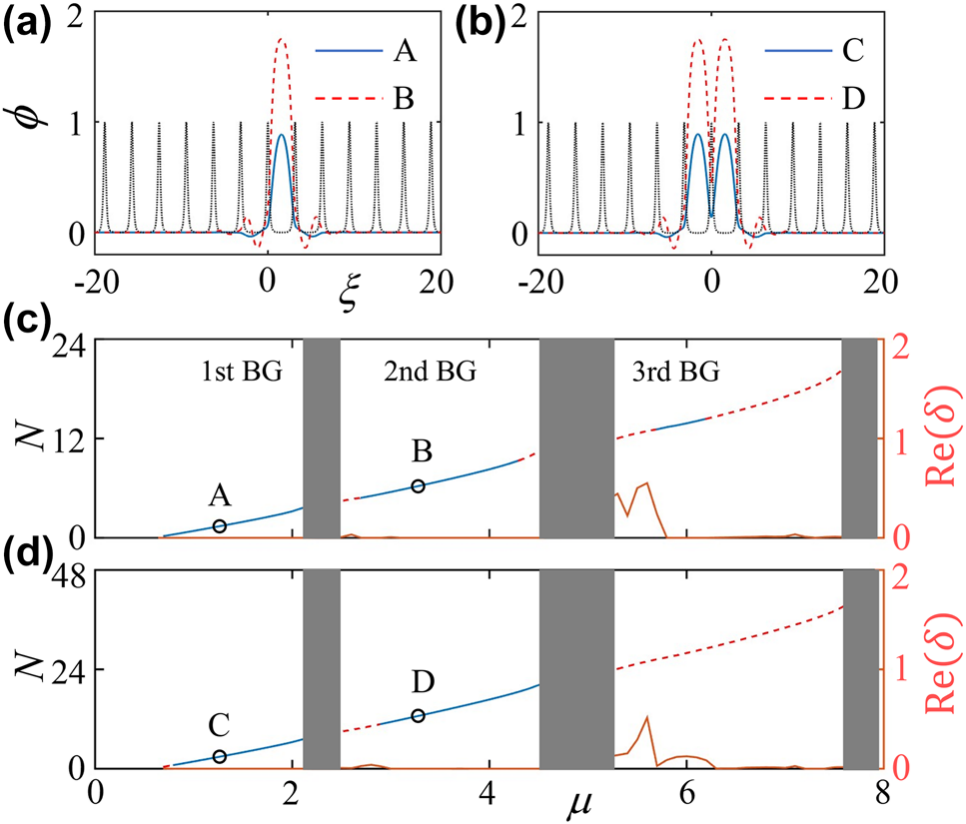
Number of atoms *N* versus chemical potential *μ*, linear-stability results, and profiles of fundamental gap solitons in a shallow lattice (*ɛ* = 0.25). Typical profiles of off-site (a) and on-site (b) gap solitons within the first and second finite gaps. The black dotted lines in (a, b) represent the normalized nanoscale dark-state lattice. Dependency *N*(*μ*) for off-site (c) and on-site (d) gap solitons, whose eigenvalues shown as the maximal real value of the perturbation growth rate Re(*δ*) versus *μ* are depicted as red solid line. Stability and instability regions for gap solitons in panels (c, d) are marked by blue solid and red dashed lines, respectively.

Below, we focus our attention on the formation of fundamental gap solitons which, in principle, can be grouped as on-site type and off-site one according to the positions of their central parts that place, respectively, at the maximum and minimum values of the nanoscale dark-state lattice. Characteristic examples of off-site fundamental gap solitons supported by a shallow lattice (*ɛ* = 0.25) are depicted in Figure 3(a), showing that such mode is single-peaked, isotropic, and modulated in a cusplike shape. The same structure properties appear to the on-site gap solitons, as displayed in Figure 3(b), one exceptional property for the on-site mode is the double-peak structure, with the spacing *D* between the two peaks equating the period of lattice. For both modes, the side-peak modulations become stronger when the gap solitons are prepared in the second finite band gap (of the underlying linear Bloch-wave spectrum). Our linear-stability analysis and direct numerical simulations of the perturbed gap solitons solutions added with initial small perturbations, both reach an excellent agreement, demonstrate three unique properties of the gap solitons: (i) the stability regions of both off- and on-site modes, created in the shallow lattice, constrain greatly from the first through second to the third band gaps; (ii) the stability region for the former is wider than that of the latter mode; (iii) in the third band gap, the stable fundamental gap solitons are only for off-site mode, as can be seen from the number of atoms *N* versus chemical potential *μ* and the underlying linear-stability results of off- and on-site gap solitons in Figure 3(c) and (d), respectively.

**Figure 4:**
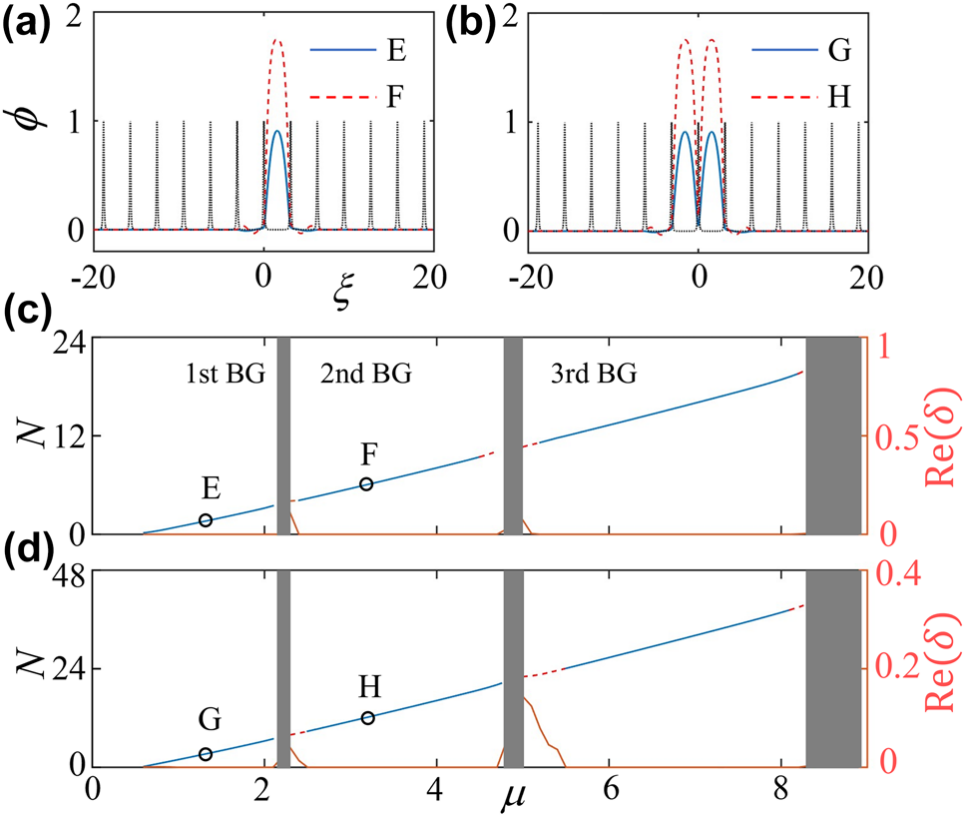
The same as Figure 3, but with a 1D deeply nanoscale dark state optical lattice with strength *ɛ* = 0.1.

For the fundamental gap solitons generated in a deep lattice, their off- and on-site modes can be constructed too, see typical examples of them in Figure 4(a) and (b). Compared to their counterparts in a shallow lattice [c.f. Figure 3(a) and (b)], the structure property remains for the case of deep lattice, a distinctive difference is the shrinkage of the spatial side modulations. The fact that the fundamental gap solitons accompany always by a cusplike modulation in the deep nanoscale dark-state OLs, challenging their general cases without any modulation in deep regular lattices (without a subwavelength structure)—which is a common knowledge [56, 57], demonstrating once again the unique feature of the localized gap modes supported by this novel type of nanoscale lattice. Such counterintuitive feature is naturally, and may be understood by taking into account the fact that, in a nanoscale dark-state lattice, the width of a subwavelength barrier is not sufficient for a complete Bragg scattering (which is balanced by nonlinearity to form a gap soliton) and, therefore, a necessary condition is the multiple Bragg scatterings which induce side modulations of a gap solitons. By contrast, in a conventional deep optical periodic potential, almost all the atoms (of a gap soliton) are within a single cell (whose width satisfies well with the Bragg resonance condition) of the lattice, making the availability of tight binding approximation (the matter-wave gap solitons are fully localized in a potential minimum cell and are entangled via tunneling to their nearest neighbors) and the discrete nonlinear equation (model) [56, 57]. Opposing to their stability regions in shallow lattice [c.f. Figure 3(c) and (d)], the gap solitons of both off- and on-site types are exceptionally stable even extending to the third finite band gap, according to Figure 4(c) and (d), uncovering the strong localization property in deep lattices. We emphasize that the dynamical perturbed evolutions of both localized gap modes in shallow and deep nanoscale lattices have been collected in Appendix B.

### 3.2 Dipole matter-wave gap solitons in a deeply nanoscale dark-state lattice

**Figure 5:**
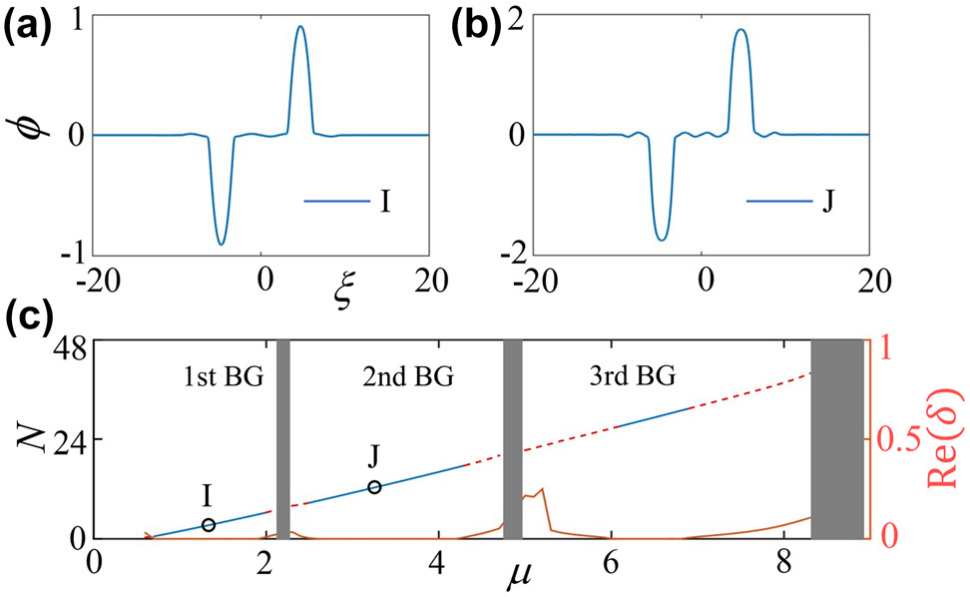
Number of atoms *N* versus chemical potential *μ*, linear-stability results, and profiles of dipole gap solitons in a deeply lattice (*ɛ* = 0.1). Typical profiles of dipole gap solitons within the first (a) and second (b) finite gaps. (c) Dependency *N*(*μ*) for 1D dipole gap solitons, whose eigenvalues shown as the maximal real value of the perturbation growth rate Re(*δ*) versus *μ* are depicted as red solid line. Stability and instability regions for the gap solitons in panel (c) are marked by blue solid and red dashed lines, respectively.

In the presence of a deeply nanoscale dark-state lattice, it is interesting to study the existence and property of compound solitons, the higher-order gap solitons, the simplest mode of which is the dipole gap solitons. It is well known that the stability region of higher-order gap solitons is always narrower than that for fundamental modes and, as shown above in Figure 3(c) and (d), whose stability in the third band gap is limited to a small region under the shallow lattice, therefore here we concentrate our interest on a deeply nanoscale dark-state lattice. Figure 5(a) and (b) display the dipole gap solitons, populated, respectively, in first and second finite band gaps, whose spacing (between the two peaks) *D* is set as 3*π* (triple to that of the lattice period). Our numerous simulations verify that the dipole matter-wave gap solitons can be stable physical objects provided that the spacing *D* ≥ 3*π*. The dependency *N*(*μ*) for such dipole gap solitons is shown in Figure 5(c), displayed in this panel is also for their linear-stability analysis by solving the corresponding eigenvalue problem in Appendix C. It is observed that the dipole gap solitons are robustly stable in first two band gaps, while have a limited stability region in the third gap where the stronger Bragg reflections demolish the phase property of the dipole mode. It should be noted that the narrow dipole (antisymmetric) gap solitons (alias subfundamental gap solitons) [54], each soliton of which is confined into a single cell, are completely unstable in the given nanoscale dark-state lattice.

Let us discuss the experimental observation of the predicted localized gap solitons in ultracold atoms systems. The first issue is to compare to the cases in conventional optical periodic potentials (with half wavelength periodic) whose shallow and deep depths contexts, as reported before, have aided to the creation of matter-wave gap solitons [50] and broad gap waves [51] in Bose-condensed ^87^Rb atoms with repulsive atom–atom interaction, with total number of atoms around 250 and 5000. Our theoretical model is quantitatively the same as the experimental situations, replacing only the conventional OLs by a nanoscale dark-state lattice [Eq. (2)]. By comparison, although the localized gap modes predicted here may be realized in the photonic crystals of “Kronig–Penney” type [52–54] which, in reality, are more difficult to make in experiments.

We remark that the previously literatures [58–60] on subwavelength plasmonic/photonic lattice solitons in arrays of metallic nanowires where the inherent loss is inevitable, and the localized modes share the same formation mechanism of graceful balance between Bragg reflections (induced by periodic potentials) and nonlinearity. In terms of experiments, the nanoscale dark-state OLs with tunable lattice depth, periodicity, and spatial structural distribution can be easily fabricated, and are a more promising platform for soliton generation and manipulation accordingly.

## 4 Conclusions

Summarizing, we have investigated numerically and with an analysis the existence, property and dynamics of nonlinear localized modes of BECs trapped by 1D nanoscale dark-state OLs with both shallow and deep strengths. The matter-wave localized modes appearing as on- and off-site fundamental gap solitons and dipole modes, prepared in the first, second, and third finite band gaps of the underlying linear band-gap structure, were demonstrated. Linear-stability analysis combining the direct perturbed numerical simulations was utilized to evaluate the stability regions of all the localized modes, and they reach a quantitative agreement. In particular, we found that the matter-wave gap solitons are always in a cusplike mode (with multiple side peaks) even being created in a deeply subwavelength adiabatic lattice, in contrast to the scenario of conventional deep optical periodic potentials where the whole gap solitons are spatially localized inside a single lattice cell (potential minimum) and thus the tight binding approximation and discrete model can apply. The effective discrete nonlinear Schrödinger equation, in essence, is derived from the continuous (mean-field) full wave Gross–Pitaevskii equation which contains complete static and dynamical details of the Bose–Einstein atoms. The predicted localized modes are highly accessible in current ultracold atoms experiments, laying a solid theoretical foundation for future experimental realization, and opening new opportunities to reveal nonlinear localized wave regimes using optical standing waves (or other periodic potentials like photonic crystals and lattices in optics) at the subwavelength.

## Figures and Tables

**Figure 6:**
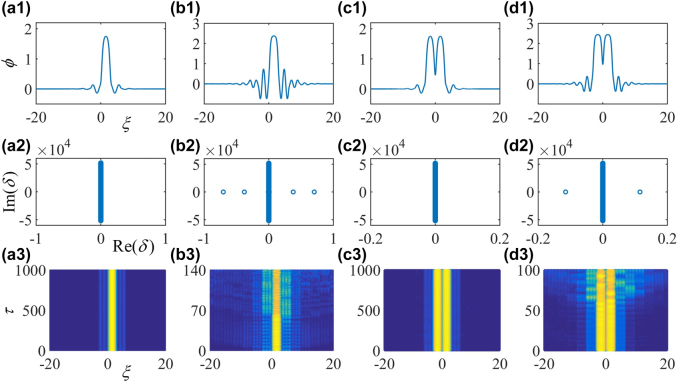
The profiles of off-site localized gap modes at *μ* = 3.2 (a1) and *μ* = 5.7 (b1), and on-site localized gap modes at *μ* = 3.2 (c1) and *μ* = 6.0 (d1) in a shallow lattice (*ɛ* = 0.25). Subfigures (a2–d2) and (a3–d3) are the corresponding eigenvalues and perturbed evolutions of the off-site and on-site gap solitons displayed in subfigures (a1–d1).

**Figure 7:**
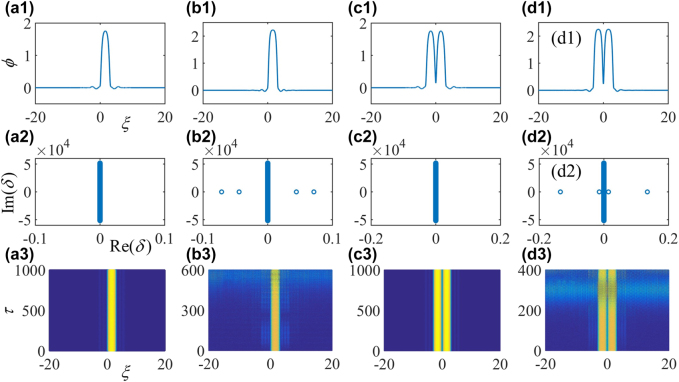
The profiles of off-site localized gap modes at *μ* = 3.2 (a1) and *μ* = 5.0 (b1), and on-site localized gap modes at *μ* = 3.2 (c1) and *μ* = 5.1 (d1) in a deeply lattice (*ɛ* = 0.1). Subfigures (a2–d2) and (a3–d3) are the corresponding eigenvalues and perturbed evolutions of the off-site and on-site gap solitons displayed in subfigures (a1–d1).

**Figure 8:**
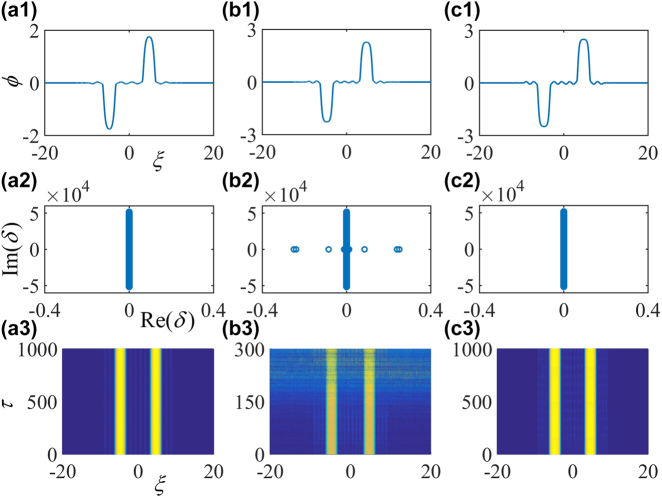
The profiles of dipole localized gap modes under changeable chemical potential *μ*: (a1) *μ* = 3.2, (b1) *μ* = 5.2, and (c1) *μ* = 6.2 with a deeply lattice (*ɛ* = 0.1). Subfigures (a2–c2) and (a3–c3) are the corresponding eigenvalues and perturbed evolutions of the dipole gap solitons displayed in subfigures (a1–c1).

## Appendix A: Linear stability analysis and numerical methods

The stationary solutions *ϕ*(*ξ*) are constructed by solving Eq. (4) by using the Newton’s iteration, and their stability property against linear perturbation is a fundamental issue. For such purpose, we take the perturbed wave function as *ψ*(*ξ*, *τ*) = [*ϕ*(*ξ*) + *p*(*ξ*) exp(*δτ*) + *q**(*ξ*) exp(*δ***τ*)] exp(−*iμτ*), here *ϕ* the unperturbed wave function calculated from Eq. (4), *p*(*ξ*) and *q**(*ξ*) are small perturbations at a defined eigenvalue *δ*. Substituting such expression into the dynamical model Eq. (3) can lead to linear eigenvalue problem:

(5)
$$\left(\begin{array}{cc} L & \phi^{2}\\ -\phi^{*2} & -L \end{array}\right)\left(\begin{array}{c} p\\ q \end{array}\right)=i\delta \left(\begin{array}{c} p\\ q \end{array}\right),$$

with $L=-\mu -\frac{1}{2}\frac{\partial^{2}}{\partial \xi^{2}}+2|\phi {|}^{2}+V\left(\xi \right)$ . In our numerics, the eigenvalue equation is solved by means of the Fourier collocation method [61], the corresponding eigenvalues *δ* decide the stability conditions of the perturbed localized mode solutions—they are stable when all the real parts of the eigenvalues are zero, i.e., Re(*δ*) = 0; otherwise, they are unstable objects. Furthermore, the solution’s stability is also measured via direct numerical simulations of the perturbed solution in the dynamical model [Eq. (3)] using the high-accurate numerical algorithm based on fourth-order Runge–Kutta method; and remarkably, outcomes of the linear stability analysis and direct perturbed simulation match very well.

## Appendix B: Dynamics of fundamental gap solitons

In this section, the time evolution of perturbed fundamental matter-wave gap solitons is displayed by using the above mentioned two ways, the linear stability analysis and direct numerical simulations.

We focus first on a shallow nanoscale dark-state optical lattice with strength *ɛ* = 0.25. In Figure 6, we have shown the profiles of localized gap solitons constructed as off-site and on-site modes (their structures and dependency *N*(*μ*) have been described in the main text) in the first line, with the stable solitons in the midst of the second finite gap and the unstable ones near the left edge of the third finite gap, their corresponding eigenvalues via the linear stability analysis are collected in the second line, and the direct perturbed dynamics are shown in the bottom line. It is observed that the direct perturbed evolutions over time indeed match up with the linear stability analysis results, and the robust coherence keeps for the stable fundamental gap solitons while the unstable solitons are subject to decay and radiation.

For the case of a deeply nanoscale dark-state optical lattice with strength *ɛ* = 0.1. The profiles, linear-stability spectra (eigenvalues), and direct numerical simulations of the perturbed fundamental gap solitons of the off-site and on-site types are collected in Figure 7, where the unstable fundamental gap modes can be existed with a longer time compared to that in a shallow lattice, and the stable solitons keep their excellent coherence over the long time evolution. The similarity of fundamental gap solitons in shallow and deeply lattices is that they are very stable in the first finite gap, and partially unstable only close to the edges of the second and third gaps, thus their perturbed evolutions are only shown when they are excited within the latter two gaps.

## Appendix C: Dynamics of dipole gap solitons

We then discuss the dynamics of higher-order gap solitons consisted of dipole mode, two off-site fundamental matter-wave gap solitons with opposite amplitudes. As described in the main text, the dipole gap solitons supported by the nanoscale dark-state optical lattice are also constructed with the spacing between the two peaks *D* = 3*π*, three times to that of the lattice period. It should be emphasized that although we here concentrate on the case with *D* = 3*π*, the stability properties can also be envisioned under the situation of *D* > 3*π*, recalling that any higher-order gap solitons should have a more narrower stability region compared to that of their fundamental counterparts, on account of the destructive interference between the solitons in the former.

As the examples shown above, in Figure 8, we also display the profiles, linear-stability spectra (eigenvalues), and direct simulations of the perturbed dipole matter-wave gap solitons of Bose–Einstein condensates trapped by a deeply nanoscale dark-state optical lattice with strength *ɛ* = 0.1. One can observe from the figure that the dipole gap solitons could generate perturbations between the interference of two solitons, enlarging their robustness against initial perturbations and making their stability regions are further compressed near the edges of the finite gaps (as also depicted in their *N*(*μ*) dependency in the main text).
